# Rigidity analysis of protein biological assemblies and periodic crystal structures

**DOI:** 10.1186/1471-2105-14-S18-S2

**Published:** 2013-11-05

**Authors:** Filip Jagodzinski, Pamela Clark, Jessica Grant, Tiffany Liu, Samantha Monastra, Ileana Streinu

**Affiliations:** 1 Department of Mathematics, Smith College, Northampton, MA, USA; 2 Department of Biological Sciences, Smith College, Northampton, MA, USA; 3 Department of Computer Science, Central Washington University, Ellensburg, WA, USA; 4 Department of Computer Science, Smith College, Northampton, MA, USA

## Abstract

**Background:**

We initiate *in silico *rigidity-theoretical studies of biological assemblies and small crystals for protein structures. The goal is to determine if, and how, the interactions among neighboring cells and subchains affect the flexibility of a molecule in its crystallized state. We use experimental X-ray crystallography data from the Protein Data Bank (PDB). The analysis relies on an effcient graph-based algorithm. Computational experiments were performed using new protein rigidity analysis tools available in the new release of our KINARI-Web server http://kinari.cs.umass.edu.

**Results:**

We provide two types of results: on biological assemblies and on crystals. We found that when only isolated subchains are considered, structural and functional information may be missed. Indeed, the rigidity of biological assemblies is sometimes dependent on the count and placement of hydrogen bonds and other interactions *among *the individual subchains of the biological unit. Similarly, the rigidity of small crystals may be affected by the interactions *between *atoms belonging to different unit cells.

We have analyzed a dataset of approximately 300 proteins, from which we generated 982 crystals (some of which are biological assemblies). We identified two types of behaviors. (a) Some crystals and/or biological assemblies will aggregate into rigid bodies that span multiple unit cells/asymmetric units. Some of them create substantially larger rigid cluster in the crystal/biological assembly form, while in other cases, the aggregation has a smaller effect just at the interface between the units. (b) In other cases, the rigidity properties of the asymmetric units are retained, because the rigid bodies did not combine.

We also identified two interesting cases where rigidity analysis may be correlated with the functional behavior of the protein. This type of information, identified here for the first time, depends critically on the ability to create crystals and biological assemblies, and would not have been observed only from the asymmetric unit.

For the Ribonuclease A protein (PDB file 5RSA), which is functionally active in the crystallized form, we found that the individual protein and its crystal form retain the flexibility parameters between the two states. In contrast, a derivative of Ribonuclease A (PDB file 9RSA), has no functional activity, and the protein in both the asymmetric and crystalline forms, is very rigid.

For the vaccinia virus D13 scaffolding protein (PDB file 3SAQ), which has two biological assemblies, we observed a striking asymmetry in the rigidity cluster decomposition of one of them, which seems implausible, given its symmetry. Upon careful investigation, we tracked the cause to a placement decision by the *Reduce *software concerning the hydrogen atoms, thus affecting the distribution of certain hydrogen bonds. The surprising result is that the presence or lack of a very few, but *critical*, hydrogen bonds, can drastically affect the rigid cluster decomposition of the biological assembly.

**Conclusion:**

The rigidity analysis of a single asymmetric unit may not accurately reflect the protein's behavior in the tightly packed crystal environment. Using our KINARI software, we demonstrated that additional functional and rigidity information can be gained by analyzing a protein's biological assembly and/or crystal structure. However, performing a larger scale study would be computationally expensive (due to the size of the molecules involved). Overcoming this limitation will require novel mathematical and computational extensions to our software.

## Background

Proteins are essential parts of all organisms. Some have structural roles, some help to mitigate enzymatic reactions; they are involved in virtually all cellular processes. Proteins are made up of long chains of amino acids joined end-to-end, whose interactions cause the chain to fold forming additional secondary, tertiary, and quaternary structures [1]. The function of a protein is often correlated with conformational changes that are driven by molecular interactions. To better understand a protein's function, its motion can be simulated and critical chemical interactions among its constituent parts can be observed. However, such simulations are computationally intense and often intractable. An alternative method is to model a protein as a mechanical structure made up of rigid and flexible parts. The overall rigidity of such a structure can be analyzed using an effcient combinatorial algorithm, which is described below, and the resulting decomposition into rigid clusters yields insights into the possible motions of the protein. Rigidity analysis has proven its usefulness [2] in giving insights about protein flexibility, but until now such studies have been performed only on a small set of proteins, each one taken in isolation.

Over 87% of the protein structures deposited in the Protein Data Bank have been solved with X-ray crystallography. Also, protein function is correlated with flexibility, and one would expect that in tightly packed crystals, some flexibility would also be lost. Indeed, in work done by Zhang, *et. al *[3], an analysis of 25 crystal forms of T4 lysozyme revealed that crystal contacts perturb a protein's backbone by 0.2 to 0.5Å. Protein flexibility studies using rigidity analysis have been performed until now primarily on *individual *asymmetric units (the smallest part of a protein that is needed to re-create the protein's biological functional form) from the data available in the PDB.

For some proteins the asymmetric unit is identical to the biological assembly. However, for many proteins, especially those determined via X-ray crystallography, the asymmetric unit is different from the biological assembly. Depending on how the protein was crystallized, the relationship between the asymmetric unit and the biological assembly can vary from protein to protein. Some biological assemblies are composed of many copies of the asymmetric unit.

The PDB file contains only the atomic coordinates of the asymmetric unit, and it is natural to expect that its rigidity analysis may not always reflect the flexibility properties of the biological form. One extreme example is a viral capsid: the icosahedral type is composed of 60 repeating units, and each one may contain several monomeric units. To gain information on the flexibility of the virus would require building the entire assembly, but so far, no existing software automatically performs this task.

**Figure 1:**
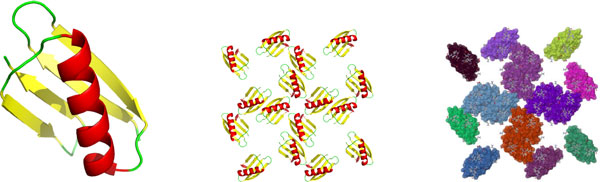
**(left) The cartoon rendering of the asymmetric unit of PDB file 2ON8 **[29]**; (center) a small crystal structure for 2ON8, and (right) its flexibility analysis results obtained by using the KINARI software**. The different colors designate distinct rigid clusters.

In this paper, we demonstrate that new insights into protein flexibility can be obtained by performing rigidity analysis on *biological assemblies *and protein *crystal structures*. These computational experiments were performed using an in-house implementation and new tools now made available in the new release of our KINARI-Web server http://kinari.cs.umass.edu, which relies on an effcient graph-based algorithm. For a proof-of-concept, we incorporated some freely available scripts for building crystals. KINARI [4] comes with tools for curating the input data and for visualizing the rigidity results. To investigate the rigidity of biological assemblies, we developed and integrated with KINARI a BioAssembly tool, that permits a user to generate biological structures from the asymmetric units typically recorded in PDB files. Figure 1 (right) shows its output of rigid clusters for a small crystal structure of protein 2ON8; different colors indicate different rigid bodies.

We tested our method on 982 crystals of various sizes, build from 324 protein structure files retrieved from the PDB. We found that the rigidity results vary among different proteins, an indication that there is additional information to be extracted from rigidity analysis of crystals and biological assemblies, as opposed to just a single asymmetric unit. In some cases, the biological unit, analyzed in isolation, exhibits significantly more flexibility than its crystal counterpart, while in others, the rigidity properties appear to be stable in the two forms.

For these 324 proteins, we build the unit cell, as well as 2x1x1, and 2x2x1 crystals from the asymmetric unit data in each PDB file. The rigidity results were analyzed to reveal trends in the rigidity properties of the crystal lattices. This indicated that chemical interactions among individual proteins in a lattice can greatly impact the crystallized form of the protein. However, some crystal structures are *not *stabilized by these additional chemical interactions, and this may reflect a biologically relevant property. Indeed, it is reported in the literature [5] that the Ribonuclease A (RNase A) protein (PDB code 5RSA) retains its function even in the crystallized form used for its X-Ray structure determination. On the other hand, a derivative of RNase A protein (PDB code 9RSA) retains only 1% of its activity, and our analysis revealed that it is very rigid, in both the asymmetric and crystal lattice forms. *The rigidity results of these two forms of RNase A correlate surprisingly accurately with their reported activities*.

We describe now the theoretical foundations of our method, the computational experiments, detailed case studies for several crystal structures and biological assemblies, and our survey of the rigidity properties of 982 crystal. We conclude with a discussion of future work.

### Rigidity analysis and mechanical models of proteins

Geometric and combinatorial methods from rigidity theory have been applied to the study of protein flexibility, by associating a network of nodes (atoms) connected by rigid bars (bonds and other stabilizing interactions). The study of rigidity and flexibility of these *bar-and-joint frameworks *dates back to a counting rule identified in 1864 by J.C. Maxwell [6]. An extension to 3-dimensional structures known as *body-bar *and *body-hinge frameworks *has been proven by Tay [7], and led to effcient algorithms [8,9] for rigidity analysis. This technique has been applied to understanding flexibility of glasses, proteins [2] and other molecular structures.

**Figure 2:**
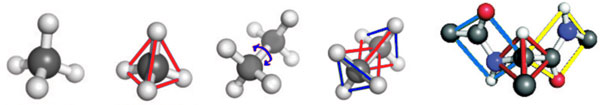
**Methane (two left images) is rigid because all pair-wise distances between atoms are fixed**. In ethane (center), a carbon atom (gray) and its bonded neighbor atoms form a rigid body. The two bodies share a hinge along the center C-C bond, shown as an abstract *body-bar-hinge framework *(second from right). A protein's peptide units are modeled as rigid bodies (far right).

In a molecule modeled as a *body-bar-hinge framework*, a **body **is a set of atoms rigidly attached to each other. For example, methane (Figure 2, two left subfigures) is rigid, because all the pair-wise distances between the atoms in this small molecule are determined by the existing covalent bond length and angle constraints. Ethane (Figure 2, center) exhibits one degree of flexibility, because the C-C bond permits rotation. Rotatable bonds are modeled as **hinges**. Two peptide units on a protein backbone (Figure 2, two right subfigures) lead to three rigid bodies, connected by two hinges.

### Rigidity analysis of protein structures

As methods for analyzing protein rigidity have advanced, rigidity analysis has been used successfully to investigate and study structural and functional aspects of various molecules. It has been found that the active sites of enzymes tend to be more rigid than other regions [10]. Rader *et al*. [11] have measured the increase in flexibility in proteins after thermal denaturation; they proposed that the folding of proteins into their three-dimensional structure be seen as a process of increasing rigidity, and verified it for the protein Rhodopsin. Protein rigidity analysis has been primarily applied to single protein units, although biomolecules and larger macromolecular complexes have been the focus of at least one recent study [12]. However, larger crystal structures have not been the subject of rigidity analysis prior to this work.

### The protein data bank, biomolecules and crystals

The proteins used in this study were retrieved from the PDB, a repository for experimental protein structure data with over 82,000 entries at this time. A PDB file contains atom coordinates for the **asymmetric unit **of a protein, i.e. the minimal set of atoms necessary to reproduce the complete protein biological assembly and crystal which was analyzed with X-ray diffraction. A PDB file has information on how to create the **biomolecule**, the functional biological unit, and **unit cell**, the repeating unit of the crystal. A **unit cell vector **is a vector from the origin of the coordinate system to a lattice point of the crystal [13]. Three unit cell vectors are needed to describe a unit cell. A symmetry operation is a transformation operation (represented as a matrix) acting on a protein that produces a copy of it, possibly translated and rotated. The **space group **referred to in the PDB file is a combination of symmetry operations and a lattice specified by the unit cell vectors.

### Generating biological units

In order to generate the biological assembly form of a protein, the asymmetric unit in a PDB file must be rotated, copied, translated, etc. The number of asymmetric units that make up a biological assembly varies from protein to protein. In some case, the asymmetric unit is the biological form, but in others, two, three, or many more asymmetric units, arranged uniquely in relation to each other, form the biological assembly. A transformation matrix in the PDB file details how chain(s) in the asymmetric unit need to be processed to form the biological form of the protein.

For the purposes of rigidity analysis, building just portions of the biological assembly may be useful. For instance, analyzing increasingly larger portions of a biological assembly can provide insight into the evolution of its flexibility as it builds up from its subunit components, or as it decomposes into its smaller subunits. From a computational point of view, generating a PDB file for the biological unit may not always be possible in the PDB format, which is what KINARI currently supports. Indeed, this format can only accommodate up to 99,999 atoms and up to 36 chains, and many large biological units such as viruses easily exceed these limits.

### Mathematics of crystallography

**Figure 3:**
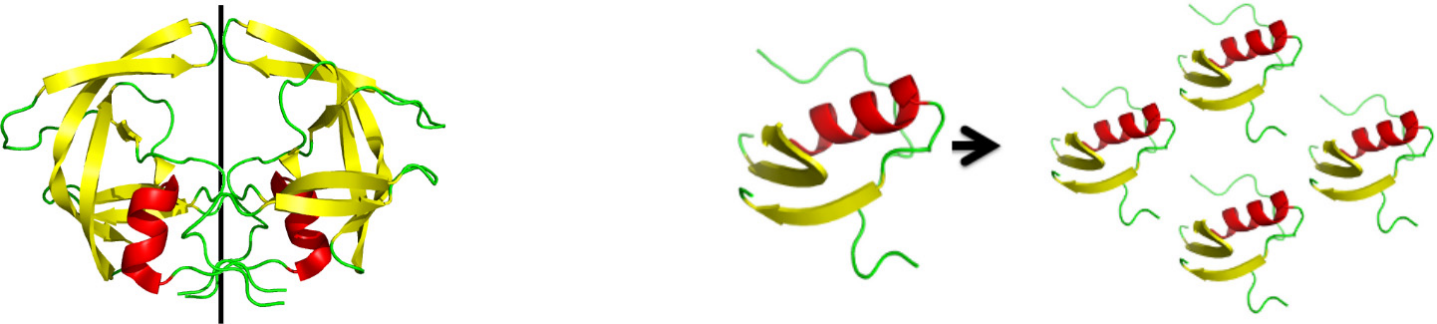
**The three symmetry operations allowed in proteins due to chirality are Rotation (left), Translation (right), and Screw Rotation (not shown)**. These operations do not compromise the handedness of the alpha-helix.

The symmetry operations, lattices, and space groups used to create a crystal from an asymmetric unit have their foundation in mathematical crystallography. There are seven types of symmetry operations, each of which has a specific matrix representation, but only three (rotation, translation, and screw rotation) are allowed in proteins due to chirality; that is, four symmetry operations are not allowed because they would change the handedness of an alpha-helix of a protein [13] (Figure 3).

**Figure 4:**
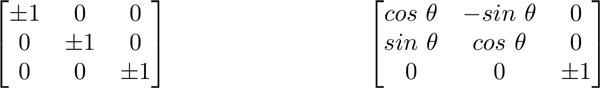
**Matrices used to generate crystal structures from the asymmetric unit can assume one of several forms, based on the specific transformation that is required to reproduce the repeating crystal units**. Depending on the combination of integer ±1 values on the diagonal (left), these can be either a rotation by 180° or a reflection. If a symmetry operation uses rotations with angles other than 180°, the matrix has the form shown on the right, with corresponding values of sin and cos substituted.

Matrix representations of two symmetry operations are shown in Figure 4. On the left, a matrix which has only ±1 values on the diagonal and zeroes elsewhere would transform the original protein data by 180° rotations and reflections about one of the three orthogonal axes. The general structure of a transformation matrix using arbitrary angles is shown on the right.

To generate a crystal, three linearly independent translations are required. If the translations are represented by three vectors, *a, b*, and *c*, then all lattice points are generated by linear combinations of the vectors with integer coeffcients [14]. There are 14 types of Bravais lattices, categorized into seven crystal groups: cubic, tetragonal, rhombohedral, orthorhombic, monoclinic, triclinic, and hexagonal.

For the purpose of this paper, a **space group **is a combination of one of the 14 lattice types and one to three symmetry operations. While there are 230 distinct space groups, proteins only crystallize into only 65 of them, due to chirality. For example, the protein in PDB file 1ONJ [15] crystallizes into space group P 4_1 _2_1 _2, with eight symmetry operations; thus it will have eight asymmetric units in the unit cell. The P and initial 4 indicate a primitive tetragonal lattice type. 4_1 _indicates a four-fold screw axis: a 90^o ^rotation, followed by a translation of 1/4 of the *c *unit cell vector length. 2_1 _indicates a two-fold screw axis: a 180^o ^rotation along with a translation of 1/2 of the *a *unit cell vector length. 2 indicates a 180^o ^rotation.

## Results and discussion

We now present three detailed case studies of the rigidity analysis of biological assemblies, which highlight why it is important to analyze a protein in its functional form as opposed to just its asymmetric unit. Then, we include three case studies of proteins analyzed in crystal form, and identify a significant, small, or no effect in rigidity. Finally, we show a survey of 982 crystal structures of various sizes, generated from 324 protein structure files.

The rigidity analysis of a protein can find a *dominant *rigid cluster, whose size is substantially larger than any other rigid cluster, or several *significant clusters *of comparable sizes. Clusters of sizes below a certain threshold are not taken into account in our analysis. We refer to them as *insignificant*. They typically belong to flexible regions.

### Merging of rigid clusters in the biological assembly - PDB structure 1HHP

**Figure 5:**
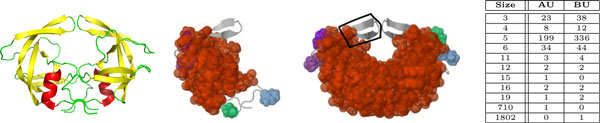
**Schematic and rigidity results of HIV-1 Protease**. The protein is a dimeric aspartyl protease (PDB file 1HHP)(left). The asymmetric unit (second from lefft) in the PDB file has one significant rigid cluster. Two of the asymmetric units make up the biological form of the protein. When the rigidity of the biological form of the protein is analyzed (second from right), the rigid clusters of the two individual monomers combine into one dominant rigid cluster. The black outlined region designates one of the two *β* hairpin loops often referred to as flaps, which function as chemical scissors and close in on the interior of the protein to facilitate an enzymatic reaction. The number of each type of rigid cluster (right) is listed for the asymmetric and biological units (AU = Asymmetric Unit, BU = Biological Unit).

As a first proof-of-concept step to demonstrate the importance of analyzing the biological assembly versus just a protein's asymmetric unit, we analyzed PDB structure 1HHP. It is the monomeric unit (one-half) of the dimer aspartyl protease, which plays a crucial function in the maturation process of HIV-1. The functional form of the protease is made up of two identical chains, each composed of 99 residues. The PDB file 1HHP contains only the asymmetric unit. Using KINARI's BioAssembly, Curation, and Rigidity Analysis tools, we compared the rigidity of the asymmetric unit in 1HHP and its biological assembly (Figure 5).

From these results, we see that the asymmetric unit of 1HHP has a dominant rigid cluster of 710 atoms, while all other clusters have 19 or fewer atoms. In the biological assembly of 1HHP, however, the two monomeric chains have a rigid cluster of approximately 1800 atoms, which is more than double the size in the monomeric unit. This demonstrates that chemical interactions between the two monomers affect the protein's rigidity. Moreover, two flap regions are identified in the biological assembly as being flexible, which is consistent with the studies on the protein's function, in which the flaps move to clamp onto the compounds in the active site of the protease [16].

### The biological assembly of a nucleoprotein - PDB file 3OUO

The Rift Valley fever virus (RVFV) nucleoprotein [17], PDB ID 3OUO, was chosen to highlight how separate domains of a structure contribute differently to the protein's overall rigidity.

The asymmetric unit in this PDB file contains a 2-chained dimer and a 1-chained monomer; each chain has 245 residues. The two biological units for this protein are the hexamer generated with three copies of the dimer and the hexamer generated with six copies of the monomer. Each monomeric chain has an extended, N-terminal arm.

**Figure 6:**
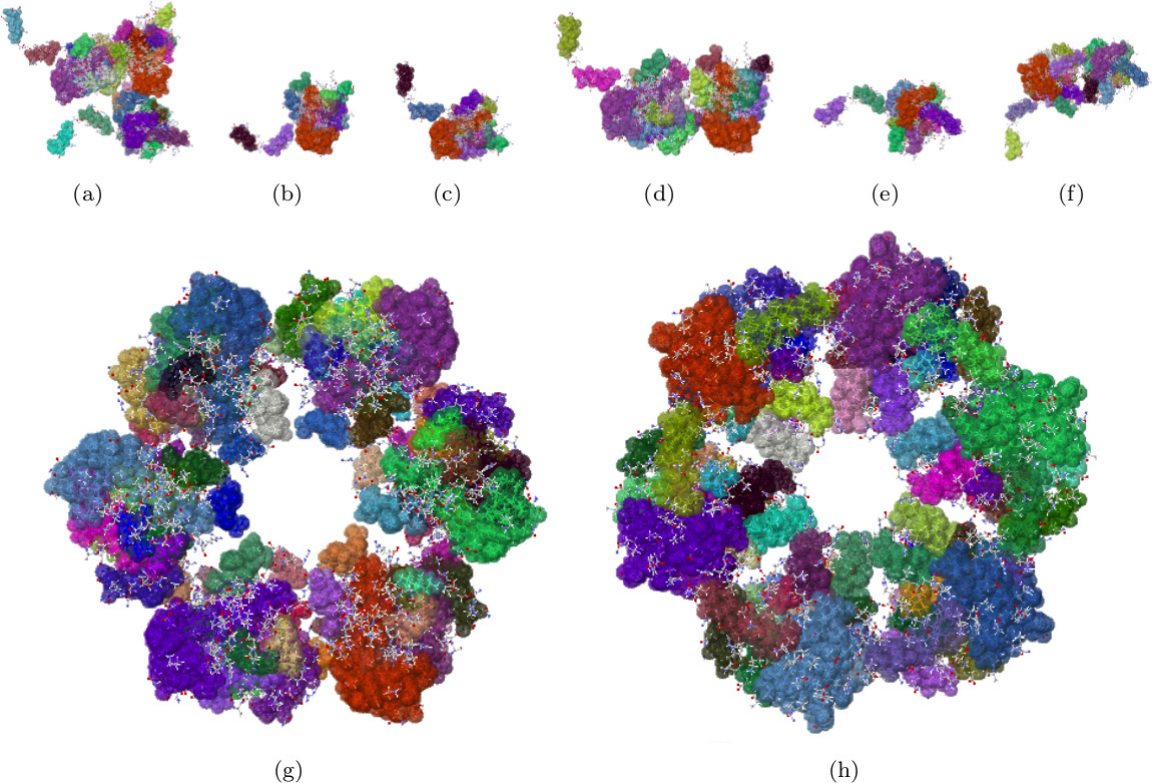
**Rigidity results of rift valley virus**. The asymmetric unit (PDB file 3OUO), is composed of three chains, A, B, and C. With the BioAssembly tool, we analyzed the rigidity of just chain A (b), chain B (c), the dimer made up of chains A and B (d), chain C (e), two copies of chain C (f), the hexamer made up of three copies of the dimer (g), and the hexamer made up of six copies of chain C (h).

We investigated the rigidity of the asymmetric unit of 3OUO, the monomeric unit of chain A, the monomeric unit of chain B, the dimer made of one copy each of chain A and chain B, the hexamer made of three copies of the A-B dimer, the monomer made of chain C, the dimer made of two copies of chain C, and the hexamer made of six copies of chain C (Figure 6). The tabulations of the rigid clusters for these components of the biological assembly show that as the structure becomes larger by a factor of *n*, the number of rigid clusters of a particular size increase by about the same factor. A closer look at Table 1 further suggests that new rigid clusters are introduced when the hexamer is built from three copies of the dimer and when the hexamer is built from six copies of the monomer. In the first biological assembly, we found three new clusters with 237 atoms; in the second, we found six new rigid clusters with 118 atoms each. These rigidity results might be explained by the fact that the N-terminal arms bind to a hydrophobic pocket in the surface of the neighboring chain of the biological assembly, which is known to stabilize the hexamer structure [17].

**Table 1:** Rigidity results for 3OUO - the number of each type of rigid cluster is listed for the asymmetric and biological unit (AU = Asymmetric Unit, BU = Biological Unit).

Size	AU	BU1a		BU1b	BU1c	BU1	BU2a	BU2b	BU2
3	215	67	66		142	454	72	148	458

4	91	33	29		62	186	30	60	182

5	1289	429	431		854	2556	435	854	2520

6	159	52	57		107	318	53	102	300

7	10	3	3		8	27	2	4	12

11	32	10	10		19	57	13	25	72

12	31	9	12		20	57	11	21	60

13	1	1	0		1	3	1	2	6

15	5	3	2		4	12	1	1	0

16	11	4	3		7	18	4	9	30

17	1	0	0		0	0	1	1	0

19	5	2	1		3	9	2	4	12

22	6	2	2		4	12	2	4	12

23	1	0	0		0	0	1	2	6

30	2	0	2		2	6	0	0	0

38	1	1	0		1	3	0	0	0

39	0	0	0		0	0	1	2	6

55	1	1	0		1	3	0	0	0

56	2	0	0		0	0	2	4	10

57	3	1	1		2	6	1	2	6

58	3	0	1		1	3	1	2	6

60	0	0	0		0	0	0	0	2

64	3	1	1		2	6	1	2	6

66	1	1	0		1	3	0	0	0

73	1	0	1		1	3	0	0	0

86	1	0	1		1	0	0	0	0

89	0	1	0		0	0	0	0	0

90	0	0	1		0	0	0	0	0

91	2	1	0		1	3	1	2	6

92	1	0	0		0	0	2	3	6

93	2	1	0		1	3	0	0	0

97	1	0	0		0	0	1	1	0

100	1	0	1		1	3	0	0	0

105	1	0	0		0	0	1	2	6

111	1	0	1		1	6	0	0	0

113	1	0	1		1	3	0	0	0

115	1	1	0		1	0	0	0	0

118	1	1	0		1	0	0	1	6

122	1	0	0		0	0	1	1	0

152	0	0	1		0	0	0	0	0

174	1	2	0		1	3	0	0	0

175	1	0	0		1	3	0	0	0

187	1	0	1		1	3	0	0	0

197	1	0	0		0	0	1	1	0

221	1	0	0		0	0	1	2	6

237	0	0	0		0	3	0	0	0

277	1	0	0		0	0	1	1	0

381	1	0	0		1	3	0	0	0

536	1	0	1		1	3	0	0	0

585	1	1	0		1	3	0	0	0

737	0	0	0		0	0	0	1	6

Column 2 is the result from analyzing the asymmetric unit, which contains one copy of A, B, and C each. Columns 3 and 4 are the results from generating only one-half of the dimer in the asymmetric unit. Columns 5 and 8 are the results from generating the dimer (chains A and B) and the monomer (chain C) in the asymmetric unit respectively. Column 7 is the result from generating two copies of the monomer (chain C). And finally, columns 6 and 9 are the results from generating the hexamer; the first from three copies of the dimer (chains A and B), and the second from six copies of the monomer (chain C).

### Analyzing how subunits of a scaffolding protein affect the molecule's rigidity - PDB file 3SAQ

**Figure 7:**
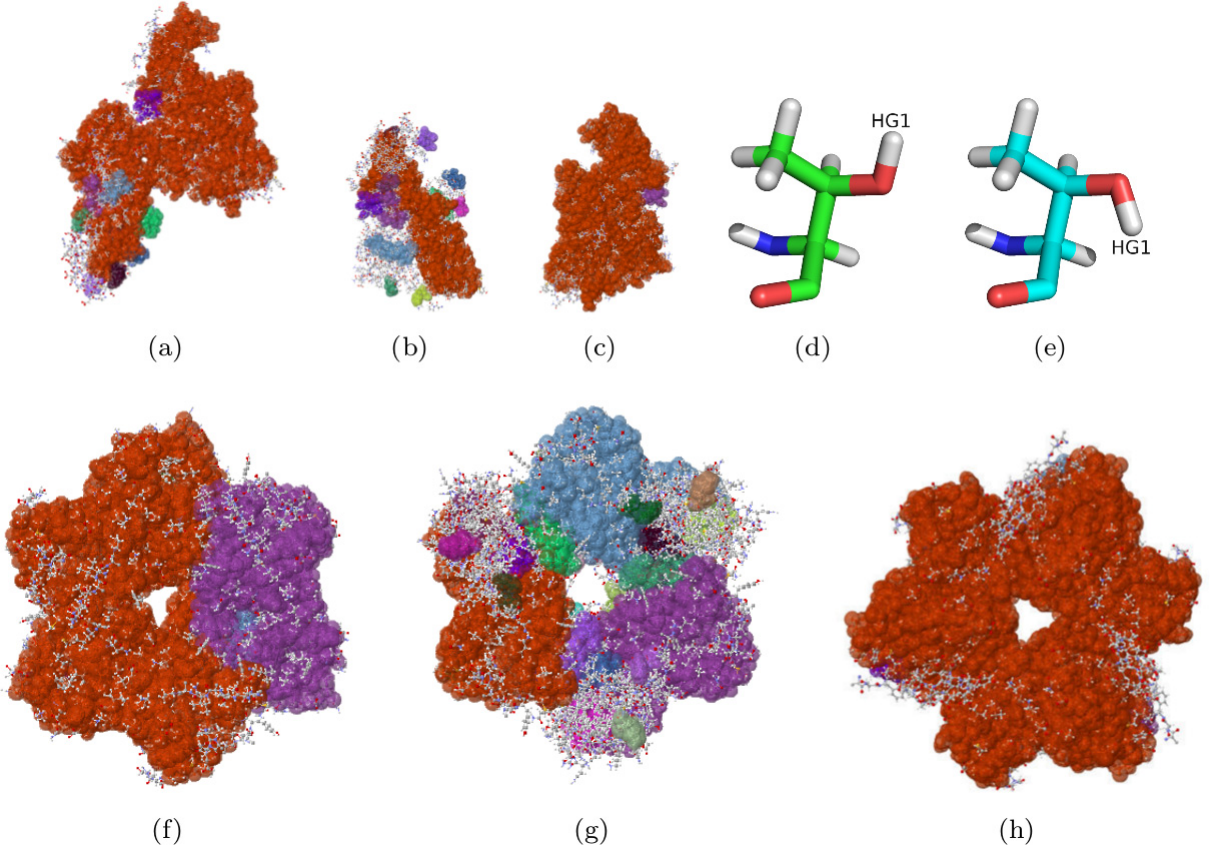
**Rigidity analysis of Vaccinia Virus D13**. The asymmetric unit of PDB file 3SAQ (a) is composed of two chains, A and B. We analyzed the rigidity of just chain A (b), just chain B (c), the biological assembly made up of three copies of chain A (g), and the biological assembly made up of three copies of chain B (f). Due to the rotamer nature of certain amino acids, hydrogen atoms were placed by the *Reduce *software at different rotamer positions for certain residues, including residue 511, Threonine (residue 511 for subunits 1 and 2 are show in (d) and (e)). This caused subunit 1 to have a different number of hydrogen bonds than subunits 2 and 3, resulting in the non-symmetric rigidity of the second biological assembly. When KINARI's curation tools were used to adjust for this discrepancy of hydrogen bonds, the resulting biological assembly was symmetrically rigid (h), as expected.

The vaccinia virus D13 (PDB file 3SAQ) is a key structural component of the outer scaffold of viral crescents [18,19]. The asymmetric unit contains two chains, A and B (Figure 7), with 576 residues each. Two biological assemblies can be generated from the PDB file. The first one is composed of three copies (subunits) of chain A, and the second is composed of three copies of Chain B.

The rigidity results for the two biological assemblies are surprisingly different, in that assembly 2, which has a dominant cluster composed of 9475 atoms, is much more rigid than biological assembly 1, whose significant rigid cluster contains far fewer 2277 atoms. To investigate this, we looked at the chemical interactions among the chains in both biological forms of the protein. Biological assembly 1 has 1092 hydrogen bonds, and 700 hydrophobic interactions, while biological assembly 2 has 1161 hydrogen bonds, and 805 hydrophobic interactions. This suggests that the stabilizing interactions among the three copies of chain B have a stronger effect on the rigidity of biological assembly 2 than do the chemical interactions among the three copies of chain A in biological assembly 1. The disparity in rigidity between the two biological assemblies might be explained by findings that multiple copies of both biological units form a honeycomb lattice, which is what provides structural stability for the immature virion membrane [19]. Thus both biological assemblies function cooperatively to perform their structural roles.

In addition, we investigated why the rigidity of the second biological assembly is non-symmetric, even though it is composed of three identical, symmetric, subunits, that are translated and rotated copies of chain B. We compared the hydrogen bonds in each of the three subunits, and found that they had 385, 386, and 384 hydrogen bonds respectively. A further inspection revealed that chain B has several amino acids, for example residue 511, Threonine, to which hydrogen atoms can be assigned in several ways. Threonine is one of two amino acids out of the naturally occurring twenty with two chiral centers [20], and it can exist as four possible stereoisomers (molecules that have the same molecular formula and sequence of bonded atoms, but that differ only in the three-dimensional orientations of their atoms in space). In addition, Threonine can assume one of several rotamer conformations even among a sample of the same protein [21,22], which explains why the adding of hydrogen atoms to such a residue can be done in one of several ways. The RMSD aligned, superimposed residues 511 for subunit 1 (Figure 7f) and subunit 2 (Figure 7g), have their HG1 hydrogen atoms (as were placed using the *Reduce *software) at different rotamer locations, which explains why one of these engages in a hydrogen bond, and the other does not.

To confirm that indeed the disparity of the number and placement of the hydrogen bonds among the three subunits of the second biological assembly is what causes the protein's non-symmetric rigidity, we performed a pairwise comparison of the stabilizing interactions among the three subunits. We identified where the three subunits differ in their hydrogen bonds. To investigate the effect of adding these hydrogen bonds involving the rotamer residues, we manually added 2, 1, and 3 hydrogen bonds to the first, second, and third subunits. We used the KINARI curation software (step 4) to insert the hydrogen bonds. In this case, the resulting rigidity of the biological assembly turned out to be symmetric (Figure 7h). Table 2 shows the counts and the sizes of the rigid clusters for the various subunits of PDB structure 3SAQ.

**Table 2:** Rigidity results for 3SAQ - the number of each type of rigid cluster is listed for the asymmetric and biological units (AU = Asymmetric Unit, BU = Biological Unit).

Size	AU	BU1a	BU1	BU2a	BU2
3	321	303	911	140	434

4	77	45	132	40	117

5	1462	905	2715	696	2117

6	179	187	561	66	217

7	1	4	12	1	4

11	16	12	36	8	25

12	45	31	93	19	56

13	2	1	3	1	3

15	2	3	9	0	0

16	2	1	3	1	3

19	7	8	24	2	7

22	1	2	6	0	0

25	1	2	6	0	0

33	1	1	3	0	1

38	1	1	0	0	0

42	0	0	3	0	0

48	1	1	3	0	0

71	1	1	3	0	0

98	2	1	3	1	3

104	1	2	6	0	1

2277	0	1	3	0	0

3912	0	0	0	0	1

4562	0	0	0	1	0

7883	1	0	0	0	0

9475	0	0	0	0	1

Column 2 is the result from analyzing the asymmetric unit, which contains one copy of A and B each, and columns 3 and 5 are the results from generating only one-third of the biological units listed in columns 4 (three copies of chain A) and 6 (three copies of chain B) respectively.

In this case study, it is striking that the placement of such a small number of hydrogen bonds in a subunit of a biological assembly can vastly alter the rigidity of the trimeric protein. In this example, adding 6 hydrogen bonds to an already existing 1161 had a profound impact on the rigidity of the biological assembly. This example corroborates with several studies that use rigidity analysis to infer the location of critical residues.

Fox, *et al *[4], have demonstrated a classification scheme identifying residues that maintain the 3D shape of a protein. In another study [23], rigidity analysis was performed in which amino acids were *in silico *mutated to glycine. This is equivalent to removing the hydrogen bond(s) in which the mutated residue engages in. This approach identified residues that could greatly disrupt the protein's rigidity when mutated, and which were conserved among several homologs.

We switch now to analyzing crystal structures.

### Dominant cluster aggregation in crystal lattice - PDB file 2YZT

The putative protein from the gram-negative bacterium *Thermus thermophilus *[24] (PDB file 2YZT), crystallizes in a P 3_1 _2 1 space group, which has 6 associated symmetry operations. Its small size (579 atoms) allows us to quickly analyze the asymmetric unit, unit cell (1x1x1), as well as 2x1x1 and 2x2x1 crystal lattices. The unit cell, the 2x1x1 crystal, and the 2x2x1 crystal have, respectively, 2, 4, and 8 asymmetric units.

**Figure 8:**
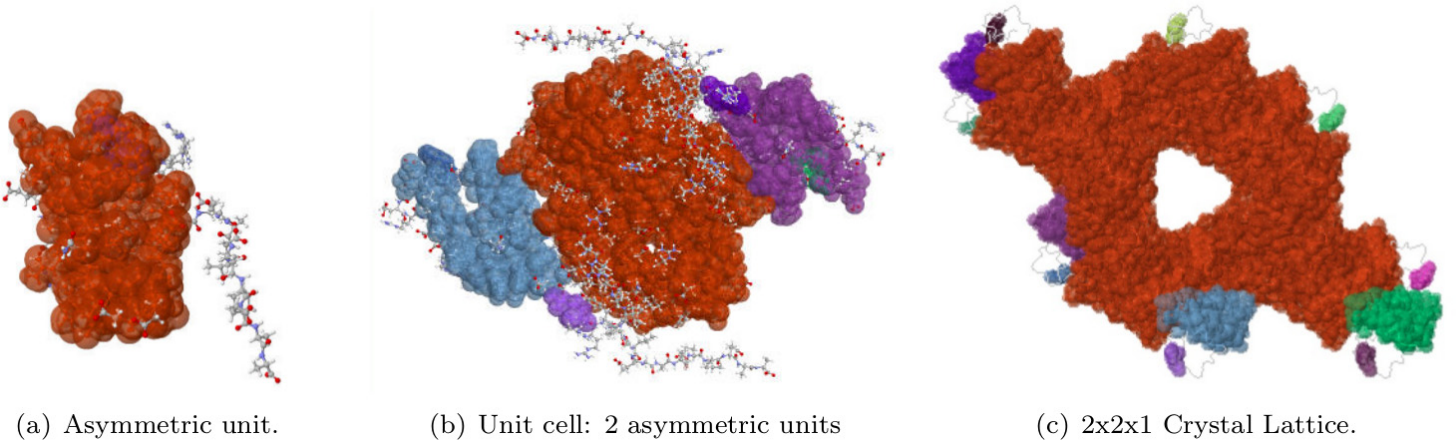
**The significant rigid cluster in the asymmetric unit (a) of PDB file 2YZT has 463 atoms**. In both the unit cell and the generated lattice, chemical interactions between the neighboring asymmetric units cause the rigid clusters of the individual units to aggregate into a dominant one.

The asymmetric unit is a globular structure with a rigid region and a tail-like segment that remains flexible (Figure 8). The dominant rigid cluster contains 463 atoms, and all other rigid clusters contain fewer than 26 atoms. The unit cell (the protein after the application of the 6 symmetry operations, i.e. the 1x1x1 crystal) maintains two significant rigid clusters of 463 atoms, but the other rigid clusters of the unit cell combine to form a rigid body containing 2504 atoms, approximately 6 times the size of the significant rigid cluster in the unit cell (Figure 8b). In other words, the significant clusters being adjacent, combine to form the dominant rigid body in the crystal. For the 2x2x1 crystal, the largest body contains 14,328 atoms (Table 3 and Figure 8c), which is significantly larger than four times the size of the significant rigid cluster in the unit cell. This indicates that the chemical interactions among the unit cells of the crystal have a significant impact on the rigidity of the entire crystal lattice.

**Table 3:** Rigidity results for 2YZT.

Size	AU	111.2YZT	211.2YZT	221.2YZT
3	4	26	49	91

4	21	106	201	308

5	122	632	1165	2126

6	22	88	133	1880

7	1	4	6	8

11	5	26	49	93

12	2	8	12	16

26	1	4	6	8

463	1	2	3	4

2504	0	1	0	0

6084	0	0	1	0

14328	0	0	0	1

The number of each type of cluster is shown for the asymmetric unit (AU, column 2), the unit cell (column 3), the 2x1x1 crystal (column 4), and 2x2x1 crystal (column 5)

### The number of significant rigid clusters increase in crystal lattice - PDB File 1UCS

**Figure 9:**
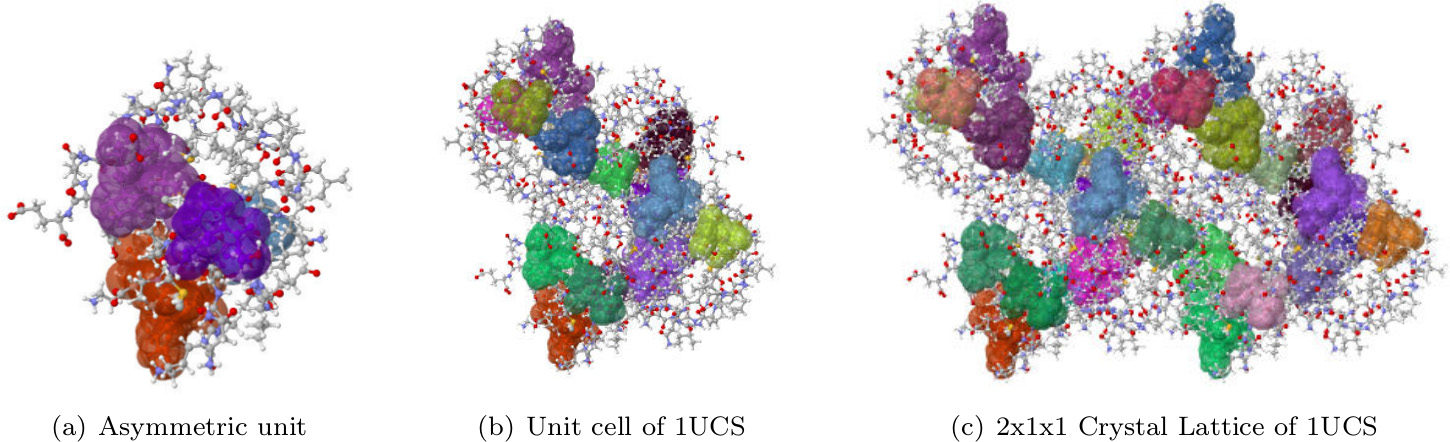
**The asymmetric unit (a) of PDB structure 1UCS is composed of four significant rigid clusters (and many more smaller ones that are not displayed)**. Unlike PDB structure 2YZT (Figure 8a), none of these is substantially larger than the others. In this case, the number of significant rigid clusters in the crystal form is more than the sum of the significant rigid clusters of the asymmetric units. This indicates interactions between the units that affect the rigidity of the crystal structure.

In the previous case study we obtained a very rigid crystal of PDB file 2LZM, with a very large dominant cluster. This is not always the case. Aggregating the asymmetric units of some proteins into a crystal lattice does not appear to greatly effect the rigidity of the resulting crystal structure. We illustrate this with PDB file 1UCS, which contains one of the four types of antifreeze proteins found in marine fish living at sub-zero temperatures [25]. This protein crystallizes in a P 2_1 _2_1 _2_1 _space group, which has 4 related symmetry operations. The unit cell of 1UCS is made up of 3 asymmetric units, the 2x1x1 crystal has 6 asymmetric units, and the 2x2x1 crystal has 12. In this case, the asymmetric unit does not have a dominant rigid cluster; it has four small significant clusters (Figure 9). Their number increases proportionally to the size of the crystal (Table 4).

**Table 4:** Rigidity Results for 1UCS: The number of each type of cluster is shown for the asymmetric unit (AU, column 2), the unit cell (column 3), the 2x1x1 crystal (column 4), and the 2x2x1 crystal (column 5).

Size	AU	111.1UCS	211.1UCS	221.1UCS
2	1	5	11	22

3	63	255	511	1029

4	8	32	64	128

5	181	718	1434	2868

6	46	189	375	747

7	3	14	30	63

8	4	16	32	64

11	1	4	8	16

12	1	4	8	16

19	3	12	24	48

23	1	3	5	10

27	1	4	8	16

36	0	1	3	6

45	1	4	8	16

67	1	4	8	16

### Rigidity analysis of Ribonuclease A - PDB files 5RSA and 9RSA

**Figure 10:**
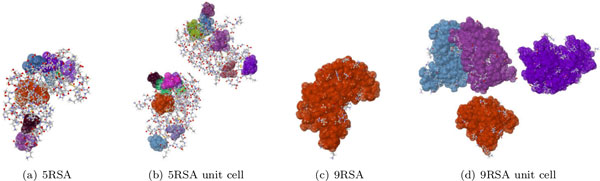
**For Ribonuclease A (PDB file 5RSA), which performs its function even in its crystalline form **[5]**, the asymmetric unit (a) and its unit cell (b) are largely flexible**. In contrast, a derivative of the protein, which is known to lose virtually all activity [26], the asymmetric unit (c) and unit cell (d) are both very rigid, and contain far more atoms in the dominant rigid cluster than the structure in file 5RSA.

Our last cast study is on Ribonuclease A, which we analyze based on two PDB files (5RSA and 9RSA). In one case (5RSA), we'll see that aggregating the asymmetric units into a crystal has no bearing on the rigidity of the lattice. 5RSA crystallizes in a P 1 2_1 _1 space group, with only 2 symmetry operations. The asymmetric unit, which contains a single instance of the protein, is made up of 2250 atoms. It has no dominant cluster, and the significant ones are made of approximately 65 atoms. The unit cell and the two crystals we analyzed (2x1x1 and 2x2x1) all have significant clusters of about the same size (65) (Figure 10). Unlike the previous two case studies (PDB files 1UCS and 2YZT), no clusters (significant or insignificant) are merged at the interface of the units when forming the crystals (Table 5). This may be because no hydrogen bonds form between the two asymmetric units in the unit cell (data not shown). We notice that only 4 new bonds appear between the cells that make up the 2x1x1 lattice, and only 8 in the 2x2x1 lattice. However, this small number of bonds does not preclude the formation of larger clusters (see case study for 3SAQ).

**Table 5:** Rigidity results for 5RSA.

Size	AU	111.5RSA	211.5RSA	221.5RSA
3	62	124	248	496

4	20	40	80	160

5	386	772	1544	3088

6	31	62	124	248

10	3	6	12	24

11	3	6	12	24

12	6	12	24	48

19	1	2	4	8

21	1	2	4	8

22	1	2	4	8

24	1	2	4	8

25	2	4	8	16

29	1	2	4	8

35	1	2	4	8

65	1	2	4	8

The number of each type of clusters is shown for the asymmetric unit (AU, column 2), the unit cell (column 3), the 2x1x1 crystal (column 4), and the 2x2x1 crystal (column 5).

*This protein is known to retain its function in the crystalline state *[5]. This may be due to its maintaining the overall flexibility even in a crystalline form.

RNase A is a widely studied protein, for which there are many structure files in the PDB. One such entry, file 9RSA, is that of a derivative, which is know to exhibit a complete loss of enzymatic activity [26]. We compared the rigidity results of the two forms. PDB structure 9RSA crystallizes in a P 2_1_2_1_2_1 _configuration, and the asymmetric unit contains two instances of the protein. The PDB file 9RSA contains two copies of RNase A. To make the comparison meaningful, we retained only a single instance of RNase A from file 9RSA. Interestingly, the rigidity results of the asymmetric unit of 9RSA has a dominant rigid cluster of 1339 atoms, in stark contrast to the significant rigid clusters in 5RSA.

### Survey of 982 crystal structures

In addition to the previous case studies, we surveyed a dataset of 324 proteins, in 982 biological assembly and crystal forms. As illustrated in Table 6, it is not biased towards proteins with only large dominant clusters. For each crystal, we tallied what percent of the structure's atoms were in the dominant cluster. We summarized the rigidity of the crystals and information concerning the dominant cluster in three groups, with dominant cluster size larger than 75%, between 50 and 75%, and between 25 and 50%. We also tabulated each crystal's number of hydrogen bonds and hydrophobic interactions. The generated crystals varied in size, the largest having 54,107 atoms (PDB file 3HON, 2x2x1 crystal). Some crystals were made up of as few as a single asymmetric unit, or as many as 48. The generated crystals varied surprisingly in terms of how many of the structure's atoms were part of the dominant rigid body.

**Table 6:** Summary of dataset for survey of crystal structures

# Proteins used to generate crystals	324
# Crystals generated using KINARI	982

Maximum number of asymmetric units in a crystal unit cell	48

Largest crystal (atoms)	54,107

# Crystals with hydrogen bonds between unit cells	776

# Structures with 25% or more but fewer than 50% of atoms in dominant rigid cluster	228 (18%)

# Structures with 50% or more but fewer than 75% of atoms in dominant rigid cluster	364 (29%)

# Structures with 75% or more of atoms in dominant rigid cluster	334 (27%)

We performed a preliminary classification of the proteins, based on the rigidity of their crystal lattices. We summarize it in Table 7. For the majority of proteins, we observed a behavior which we call *dominant cluster aggregation at all levels *(**case 1**). This means that the crystal contains a dominant cluster of size more than the sum of the dominant clusters in its unit cells or asymmetric units. For 27 of the proteins (**case 2**), we observed a *dominant cluster aggregation at the unit cell level*, and no aggregation at the crystal level. **Case 3 **(33 proteins), shows no change in rigidity among the asymmetric unit and any of the generated crystals. For twenty of the proteins (**case 4**) the unit cell and 2x2x1 crystal had rigid bodies that spanned several asymmetric units or unit cells, respectively, but the 2x1x1 crystal had rigid bodies that were no bigger than the rigid bodies in the unit cell. This may be because the interactions between the unit cells along one crystal lattice axis may be different. The 10 proteins in **case 5 **had asymmetric units and unit cells that had the same sized dominant cluster, but for the 2x1x1 and 2x2x1 crystals there was a collapse of the dominant cluster. **Case 6 **contains those proteins with a dominant cluster at the unit cell level that spans multiple asymmetric units but does not aggregate in the larger crystals.

**Table 7:** Preliminary Classification of Proteins According to the Rigidity Properties of Their Crystals

	Case	Num. Proteins	% of Dataset
1.	Dominant cluster aggregation at all levels	192	59

2.	Dominant cluster aggregation at the unit cell level	27	8

3.	No combining of rigid bodies in unit cell nor in larger crystals	33	10

4.	Rigid bodies of asymmetric units combined in unit cell and 2x2x1 crystal, but not for 2x1x1 crystal	20	6

5.	Size of dominant cluster in asymmetric unit and unit cell was the same, but there was aggregation of dominant body in 2x1x1 and 2x2x1 crystals	10	4

6.	Dominant cluster at unit cell that spans multiple asymmetric units but does not aggregate in crystals	24	8

7.	Other; unclassified.	18	5

There were some proteins which did not quite fit into any of the six cases. We observed that the asymmetric unit and unit cell of 2BF9 had no change in rigidity, but when building the 2x1x1 crystal there was a collapse of rigidity, and the 2x1x1 and 2x2x1 crystals had the same rigidity. For the analyses of 6RXN, the asymmetric unit, unit cell, and 2x1x1 crystal all had no additional collapse, but when the 2x2x1 crystal was built the size of the largest rigid cluster increased. Although this survey is by no means comprehensive, it already displays patterns of rigidity properties for protein crystals that motivate future extensions of our software for fully understanding protein crystal lattices.

## Conclusion

The rigidity analysis of a single asymmetric unit may not accurately reflect the protein's behavior in the tightly packed crystal environment. Using our KINARI software, we demonstrated that additional functional and rigidity information can be gained by analyzing a protein's biological assembly and/or crystal structure. However, performing a larger scale study would be computationally expensive (due to the size of the molecules involved). Overcoming this limitation will require novel mathematical and computational extensions to our software.

For the analysis of larger assemblies of asymmetric units, we found that relying on "black box" software has to be taken with a grain of salt. In the case of X-ray resolved structures, the PDB files do not contain hydrogen atoms, and these have to be placed with software (such as *Reduce*). Conversely, using the KINARI curation feature allows one to formulate and verify hypotheses concerning the molecular model, when the placement of atoms or stabilizing interactions needs to be disambiguated.

In the study of crystal structures, we found that some lattices contain rigid bodies that span multiple unit cells, while in other cases, unit cells of crystals retained the rigidity properties of their asymmetric units, in that the rigid bodies did not combine in the larger (crystal) structure. Finally, some crystals were found to be largely flexible, because the unit cells do not have stabilizing interactions among them. In summary, this work shows that rigidity analysis of protein crystals is feasible and has the potential of correlating to important function-related aspects of the protein.

For future work, we plan to focus on very large PDB files. They significantly slow down the computation time in building the molecular model, identifying chemical interactions, and performing rigidity analysis. To address these shortcomings, one might take advantage of the symmetry and periodicity in the biological units and crystals. Doing so might preclude from having to compute the locations of all atoms and interactions among symmetric portions of molecules with large symmetries and periodicity.

## Methods

Our computational setup involves the following: we parse the input PDB file, and use the information in it to build the biological unit and desired crystal structure. Then, we apply our KINARI-Web software to place hydrogen atoms, identify chemical interactions, and perform rigidity analysis, which outputs the rigidity clusters. To perform the experiments, we selected a dataset based primarily on protein size, for reasons having to do with the limitations of the current implementation. However, we emphasize that these experiments demonstrated that our software is able to handle relatively large protein structures.

**Figure 11:**
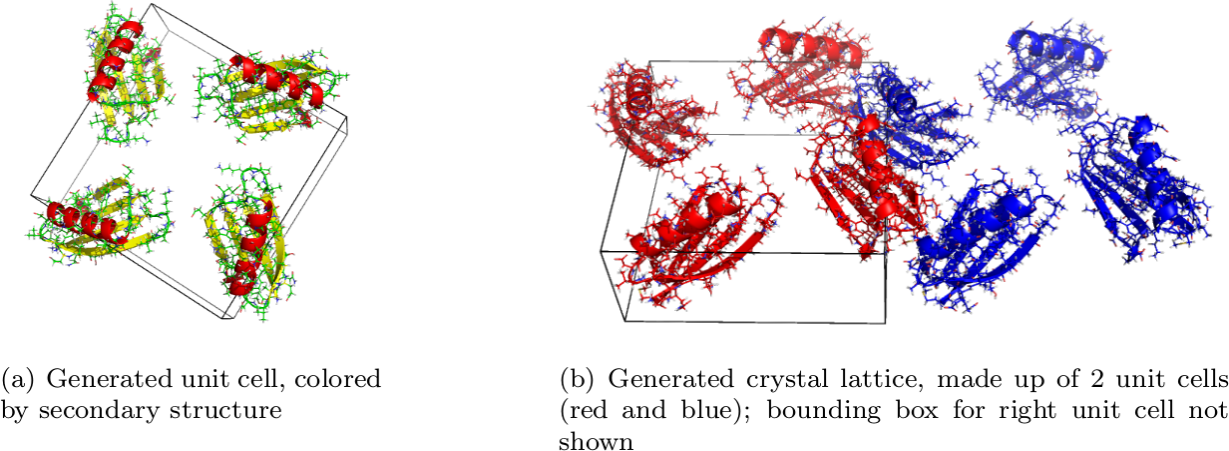
**We generated the unit cell (a) from the asymmetric unit of protein 2ON8**. In (b), the unit cell is translated using the unit cell vectors to generate a 2x1x1 crystal lattice.

To create the asymmetric unit and crystal lattices, we applied symmetry operations that were given in REMARK 350 of each structure file on each atom of the asymmetric unit. We built the unit cell with *supercell.py *[27], a Python script. It retrieves the three unit cell vectors from the CRYST1 line of a PDB file, and generates the unit cell by applying the space group symmetry operations on the asymmetric unit. We built the crystal by translating the unit cell in the direction of the three unit cell vectors (Figure 11). For self-checking, we generated the crystal structures using two methods: a custom-built interface to the python script *supercell.py *[27], and an in-house implementation. An example, illustrating the Immunoglobulin G-binding protein G (PDB file 2ON8) is shown in Figure 1 (left).

Table 8 summarizes the steps of our experimental setup and methods for generating and analyzing crystals build from PDB structure files. The advanced search feature of the PDB was used to select proteins that had between 50 and 150 residues, whose structure was determined using X-Ray crystallography. Only proteins with no DNA nor RNA were selected.

**Table 8:** Summary of Experimental Setup

Step	Description
Selection and Curation	324 PDB files selected for analysis

Building of Crystal Lattice	*supercell.py *and custom scripts were used to apply symmetry and translation operations on each protein's asymmetric unit

Performing Rigidity Analysis	The KINARI software was used to calculate the rigid regions of the asymmetric unit and crystal lattices of each protein

Identifying Rigid Clusters	The rigidity results were analyzed to determine if interactions of unit cells led to larger rigid clusters.

For generating a protein's biological assembly, we used the translation and rotation matrices included in the header of a PDB file. We applied transformation operations that were given as matrices in REMARK 350 of each structure file on each atom of the asymmetric unit. Each matrix contains a 3D rotation matrix and three translation vectors (one for each axis). The listing of secondary structures also was updated with references to the newly generated atom coordinates.

## Competing interests

The authors declare that they have no competing interests.

## Authors' contributions

IS conceived the research and supervised the project. TL implemented the BioAssembly code, PC and JG the Crystal part, and FJ supervised the code development and performed the KINARI integration. The preliminary experiments, case studies and the survey were done by TL, PC, JG and SM. IS and FJ analyzed the results and wrote the paper.

In this article we present a methodology and experimental computational results that extend our previous work. In the earlier version that appeared in an extended abstract, we reported on rigidity experiments of a limited number of crystal structures. In this work, we introduce and demonstrate KINARI's new feature of generating biological units, and we analyze their rigidity. This article also introduces the crystal and biological unit tools that are freely available on the KINARI website.

## Declaration

Publication of this article was supported by DMS-0714934, NSF CCF-1016988 and DARPA 23 Mathematical Challenges grants of IS.

This article has been published as part of *BMC Bioinformatics *Volume 14 Supplement 18, 2013: Selected articles from the Second IEEE International Conference on Computational Advances in Bio and Medical Sciences (ICCABS 2012): Bioinformatics. The full contents of the supplement are available online at http://www.biomedcentral.com/bmcbioinformatics/supplements/14/S18.

## Relationship with prior work

This is an expanded publication of the article "Periodic rigidity of protein crystal structures" [28] that was presented at the 2012 IEEE 2nd International Conference on Computational Advances in Biological and Medical Sciences (ICCABS). In this work we perform an analysis of many more crystal structures than were analyzed in the ICCABS publication. Here we also introduce several new features of the KINARI software, including the ability to create biological units from the asymmetric unit in a PDB file. The data for the three case studies of the rigidity analysis of biological assemblies presented in this publication was generated using these new KINARI tools, which are publicly available.
